# Account of the Typhus Fever, and Fatal Epidemic Variola and Varioloid Disease, That Prevailed in Halifax, Nova Scotia, in the Summer of 1827 and Winters of 1827-8

**Published:** 1829-07

**Authors:** Wm. Donnelly

**Affiliations:** Surgeon of his Majesty's Ship Hussar.


### TYPHUS AND VARIOLA.

*Account of the Typhus Fever, and fatal Epidemic Variola and Varioloid Disease, that prevailed in Halifax, Nova Scotia, in the Summer of* 1827 *and Winters of* 1827-8. By Wm. Donnelly, m.d. Surgeon of his Majesty's Ship Hussar.

The summer of 1827 and winter of 1827-8, were seasons of great sickness and mortality in the town of Halifax, arising chiefly from a wide-spreading typhus and a particularly fatal epidemic variola.

Both these diseases were introduced by emigrants from Ireland, three shiploads of whom arrived during the summer from Waterford, where they had been collected by the advertisements and agents of a Mr. Cook, to be transported *en masse*, men, women, and children, without a surgeon; and to be thrown, destitute of every thing, on the charities of a province to which they brought disease and death.

Typhus demanded the earliest attention. This disease had existed sporadically, but was rare, when, in July 1827, after the arrival of a second ship with emigrants, it began rapidly to spread through every part of the town, so that the poorhouse could not contain the cases requiring admittance, and it became necessary to establish a fever hospital. A barn belonging to a farm, called Bankhead, about a mile from town, was appropriated to this use.

To the helpless and destitute it was a great felicity to be admitted to this, the best accommodation that at the time could be afforded; yet it was a very defective asylum of disease. There were no windows, and light, air, nurses, patients, and physicians, entered by the capacious folding doors of the barn; the sick reposed on straw beds laid on the earthen floor, which were arranged closely together round the walls and in the middle of the floor; the bedclothes were scanty. Medical attendance was gratuitous, but given with commendable diligence: and food, medicines, wine, &c. were furnished by the poor-house.

The above circumstances it is necessary to bear in mind, in reference to the results of the disease. The fever hospital was established with the hope of arresting its progress in town, and consequently patients were received from the emigrant ships, the town, and poor-house. Of those admitted, a few proved to be variola, and several only ephemeral fever. Two hundred and eighty only of the three hundred and sixty-one patients that were admitted from the middle of July to the middle of October, (the three months the house was open,) were really typhus; and of these, there died at Bankhead sixty-one, and thirty were sent to the poorhouse, on the hospital being shut up. Of the latter, fiteen were convalescent, and fifteen confined to bed, but likely to recover. So that, out of two hundred and eighty cases of typhus, sixty-one, or one in little more than four and a half, may be considered the proportion of mortality.

Men, women, and children, were received as patients at Bankhead. The number of the first was equal to that of both the latter. The proportionate mortality amongst the men was little more than that amongst the women; but the number of deaths of children, in proportion, was little more than half of that amongst either women or men.

The contagious nature of the fever was evinced by its being communicated to many of the inmates of the poorhouse, who resided there from old age, or from their being afflicted with chronic or other complaints, shortly subsequent to the emigrants being first admitted from the ships, before Bankhead was appropriated to the fever patients. After this the poor-house did not get free from it for months, but sent cases from time to time to Bankhead; some of them the nurses and servants of the establishment. Also, again, on the convalescents returning to the poorhouse in October, typhus spread in quick succession amongst several of the poor there.

About the 20th October, a young man was two days in the house for an ulcer on the leg. His bed, during this time, was next to that of a typhus patient. Three weeks after, I saw him carried into the house with severe typhus fever. Of the nurses who attended at Bankhead, five caught the fever, and recovered, some of them with difficulty; and, of the orderly men, two were seized, both of whom died.

As late as the middle of December, there were six bad cases of typhus in the poor-house. Here the disease proved far less fatal than at Bankhead. It ran through the whole of ninety-one children, who were in the house at the time, with only one death, though some of the cases were severe. This great difference in mortality is rather owing to the greater mildness of the fevers and the superior comforts of the house, than to any difference in the medical treatment; as, neither at Bankhead nor in the poor-house, did the cases seem to admit or to receive generally any systematic treatment, by bleeding or other particular remedy.

Many of the typhoid patients became completely yellow, and merited to be called icterodes, or, as far as colour has procured the title, yellow fever. Four such cases occurred also in the military hospital, and many in private practice; but these did not generally prove more fatal than the other typhoid cases.

The whole mortality from typhus during this epidemic, including the fever hospital and poor-house, amounted to between two and three hundred; and this was only an item in the general mortality amongst a population of from twelve to fourteen thousand. During the eight months from June to January (both months inclusive), between a fifteenth and sixteenth of the whole population died; it appearing from the account kept of the burials, that, in the first ten months of 1827, there were 814 deaths, though this included only five of the sickly months, and the last three continued to be as fatal as either of these.

No account being kept of the diseases which cause the deaths, the numbers from each cannot, with any certainty, be assigned; still there is no doubt the majority were victims to variola. The poor-house books furnish the best criteria for estimating as well the general mortality at the time, as that particularly from smallpox. The number of deaths in this asylum annually average from fifty to sixty-five, and 105 in 1826 was a mortality unequalled for years. In 1827 there were 248 deaths, not including the sixty-one at Bankhead. The number of cases of smallpox, all admitted from the middle of June, 1827, to the 10th March, 1828, was seventy-nine; of these, forty-one recovered, and thirty-eight died, being nearly a half.

In 1820 variola appeared in Halifax, but only fifteen cases then occurred, in consequence, it was believed, of the surgeons dividing the town into districts, and each gratuitously vaccinating that division to which he was appointed.

Unaccountably, when, in June 1827, this disease again presented itself, it attracted no such attention either of the physicians or magistrates, but was allowed to proceed uninterrupted by any general measure of prevention for six months. Long ere this every part of the town was thoroughly infected, and the contagion was propagated to other vicinal parts of the province. At length the immense mortality aroused the magistrates, and about the middle of December a census was taken, the town being divided into twelve districts, and one assigned to each magistrate, to make at every house the inquiries with which he was furnished.

Some, I believe, executed this gratuitous labour with proper accuracy; but others, I know, were satisfied by calling at every door, and putting the questions to the person who first presented himself.

The only result derived from the aggregate of the numbers is, that in Halifax 2146 had been vaccinated; 1253 had had the smallpox in the natural way; 315 had neither had the smallpox nor been vaccinated; 50 had died of smallpox; 558 had had smallpox after vaccination; and 25 had died of smallpox after vaccination.

The good resulting from the census was a pretty general vaccination of all that remained unprotected, the surgeons performing this operation gratis to those that came; and to this may certainly in some degree be attributed the decline, in about a month after, of the epidemic, which had proceeded uninfluenced by the heat of summer or the cold of winter.

The facts observed during the epidemic are remarkable and interesting in the history of variola and vaccinia.

It may be well to premise, that, in the early part of summer, rubeola had prevailed to a considerable extent, and scarlatina in a minor degree; but, as the variolous epidemic advanced, these almost disappeared: and that, with regard to varicella, practitioners of Halifax say they hardly recollect a year to have passed of the last twenty without its being abundant, yet it was never known to originate variola. Indeed, except in 1820, as already mentioned, smallpox had not been in the town for twenty years.

In November 1827, varicella ran through the families of three officers, two of these physicians. In one family there were three children, one aged six, one three years, and the other seven months. In the eldest it appeared a fortnight earlier, a vesicular eruption, the vesicles distinct and widely separated, accompanied by slight pyrexia and general illness. After the third day the eruption dried away.

In the second there was a (not numerous) distinct vesicular eruption on the face, neck, and chiefly the back; the vesicles small, containing a limpid fluid, and based by the natural coloured (not inflamed) skin. The eruption was accompanied by feverishness, general illness, inappetency, and white tongue. On the third day the vesicles began to dry.

Three days later, the youngest child was more thickly covered with a similar vesicular eruption, from which, on being punctured, a limpid watery fluid exuded. On the third day, the vesicles began to dry on the face, yet on the fourth day some came out on the back. The latter did not dessicate before the sixth day. On the first, but chiefly on the second and third days, (especially at night,) this child was ill, feverish, and very fretful.

All the physicians who saw the disease just described unhesitatingly pronounced it varicella, and all the practitioners of Halifax, with whom I have conversed, state the dessication on the third day, and the little previous indisposition, as sufficiently diagnostic, even neglecting the other characteristics of the eruption.

With this disease primitive variola cannot possibly be confounded, and almost as little can its modification, the varioloid disease. This is characterized by its happening subsequently to natural or inoculated variola, or to vaccinia; by there being no secondary fever, and by the primary fever being generally less severe than that of variola; by the dessication of the pustules on the sixth day; by elevations, and not pits, remaining after the crusts fall off; by the absence of the peculiar variolous effluvium; and, lastly, by its comparative mildness.

In most cases such distinctions held; yet in many the disease, though subsequent to vaccinia or previous variolation, ran the regular course of variola, was severe, and in some even fatal; so that the previous disease did not invariably give occasion to the modification.

The facts unquestionably ascertained during the epidemic are, first, the occurrence of the modified variola (varioloid disease) after perfect vaccination, whether recent, more remote, or renewed, after perfect variolous inoculation, and even after previous accidentally contracted smallpox. Secondly, the occurrence of variola unmodified, consecutively to vaccinia and to variolation, whether from inoculation or smallpox accidentally contracted. Thirdly, of deaths from variola subsequently to perfect vaccinia, and to inoculated or accidentally acquired variolation. Fourthly, of legitimate variola from varioloid contagion; and vice versa.

Perfect secondary vaccinia was frequently witnessed, the anxieties of parents leading them very commonly to repeat vaccination, for greater security to their children. On these occasions the surgeon often saw as perfect a vaccine disease as he had originally witnessed in the same subject. One surgeon estimated that not more than one in twenty took it the second time, but other observations induce me to believe that this is fewer than was generally the case.

Mrs. U., thirty years of age, who had contracted the smallpox when young, went through perfect vaccinia, feeling even generally indisposed for two days. Two other ladies went regularly through the vaccine disease, though they had had the smallpox from inoculation. Miss J., aged fifteen, was vaccinated a few hours after her birth, (variola at the time prevailing,) and the operation succeeded so satisfactorily, that lymph was taken, to send to a distance. At six years of age she went through a varioloid disease, which was attended by the same characteristics as the present; and, during this epidemic, she had from vaccination so perfect vaccinia that I should not hesitate to say that by it alone she would be satisfactorily protected.

After smallpox had been introduced into the poor-house, and had spread extensively amongst the children, those who remained unprotected were vaccinated; some whilst inhabiting rooms nearly full of smallpox. In two I witnessed the co-existence of variola and vaccinia. One, on the eighth day of vaccinia, had the vesicle perfect, and at the same time a varioloid eruption, promising (as it proved) to be a very mild disease. In the other, the eighth day of vaccinia was the fifth of varioloid eruption; and the latter was distinctly pustular, some of the pustules dessicating; whilst the former also was pustular, very large, apparently not cellular, the vesicle seeming modified into varioloid.

I am not aware that, with regard to secondary vaccinia, vaccinia after inoculation, or variolation, and co-existent variola and vaccinia, numerous observations were made; but, respecting most of the facts previously stated, there is abundance both of popular and medical evidence.

First, of variola and varioloid disease after vaccinia. Here the public opinion was correctly and feelingly expressed in the following paragraph of a Halifax newspaper: "Opposed to the opinion of those who consider vaccination as a protection, is the melancholy condition of the town at the present moment (in December). Innumerable are the instances, and fatal instances, of failure of the vaccine inoculation in resisting the smallpox. We need not point to any particular family for proofs of this assertion: it cannot be denied." Nor did any medical man attempt to deny it. Those to whom a proportionate number of the cases now alluded to did not occur were strongly disposed to attribute the ineffectual protection to imperfect or careless vaccination; but, before the end of the epidemic, these also were forced to confess that variola or varioloid disease had been witnessed by themselves, after their own pronounced excellent, and even recent, vaccinia. Not less than 130 such cases occurred to three of the principal practitioners. A proportionate number could not (simply from their more limited extent of practice,) have happened to all the other six or seven surgeons; so that the number stated in the census must be too high.

In many cases the surgeon could not verify, by the scar, the accounts of the parents, or in any other way, the genuineness of the vaccinia said to have preceded. Some, indeed, particularly those at or beyond the age of maturity, stated that the vaccination had been by women or countrymen. Yet certainly, in the majority, conviction of the genuineness of the vaccinia could not be refused; the proof being derived from the registers of the practitioners, their own recent recollections, the perfect scar, the parents' circumstantial accounts, &c.

In the army, all the men and children are carefully vaccinated, and regularly registered by the medical officers, of whose competence there cannot be a doubt. Yet the regiments quartered at this time in Halifax did not quite escape; though, amongst 1100 men belonging to three regiments, not more than a dozen cases occurred, and, except one, all were cases of mild varioloid disease, and all of them recovered.

Amongst the children of each regiment, the disease spread more. In one, ten cases occurred in a fortnight; and all these children had been vaccinated by army medical officers. Three of them assumed the variolous character, and two died. One of the latter had been vaccinated about three years before, by the assistant surgeon of the regiment. The other became ill on the thirteenth day of good vaccinia, and died on the eleventh day of the variolous eruption. It is worthy of mention, that a child, three months old, which was vaccinated at the same time as the last child, slept with this one and another ill of variola, and escaped entirely the disease.

Generally speaking, the disease after vaccination assumed the varioloid appearance, and usually its symptoms were so extremely mild as only slightly to indispose the patient, particularly after the invasive fever; and, even where this had been sharp, the patients afterwards almost always walked about, confined to the house only by the unseemliness of the eruption, and as a caution.

The severe and fatal cases subsequent to vaccination assumed the regular and unmodified characters of variola. These formed a small portion of the whole of the cases that occurred after vaccination; one in twenty-two and a third, according to the census. But, as the numbers of varioloid disease after vaccination are to be reduced as many as might fairly be allowed for imperfect and nonprofessional vaccinations, (an inquiry not sufficiently entered into by the magistrates,) so a deduction must be made from the fatal cases for previous unsatisfactory vaccination. Of seven fatal cases of which I knew, the perfect vaccination of four only could be verified.

It was difficult to form an estimate of the number of persons vaccinated who took the disease. Some practitioners said one in ten, others one in twenty, and others one in four; each judging according to what had happened in his own practice. Of a whole family vaccinated, part would take the disease, and the others resist the concentrated contagion. When faith in vaccination had become shaken by the frequent consecutive occurrence of variola, there was some interest in finding out those who were proof against the apparently almost irresistible contagion. These, however, were very numerous, and some were remarkable instances of protection by vaccination, even when instituted subsequently to exposure to variolous contagion. One of these was that of an infant vaccinated just after birth, the mother being delivered whilst labouring under variola, of which she died. And here it may be stated, that, generally, every parturient woman having variola died, and every pregnant one aborted.

Before alluding to the occurrences of varioloid disease after variolous inoculation, it is necessary to state that vaccination had been practised in Halifax about twenty years, and that it was very generally received; at least, there appeared to be no prejudice in the minds even of the less informed against it; and that, consequently, inoculation, for not less than ten or fifteen years, must have been rare, and those protected by it, compared with the vaccinated, very few.

Notwithstanding this, not less than between forty and fifty cases of variola or varioloid disease occurred during the epidemic, secondary to inoculation or accidentally contracted variola; and these generally were more severe than the disease consecutive to vaccinia. Indeed, one of the earliest deaths that happened was from a malignant variola in a woman, seventy years of age, who had been inoculated thirty-five years before, and that effectually, as was evident to every one by her being remarkably pitted. In the perfect confidence derived from this, she freely and voluntarily exposed herself to the contagion. In December, a man of colour died of smallpox, though he had previously had the disease; and there is little room for doubt that it was of secondary variola a child, three years of age, died about the same time, on the tenth day of the disease.

This child had gone through the primary attack only six weeks before. When, on the occurrence of the invasive fever of the secondary disease, suspicion of smallpox was entertained, the surgeon was shown several distinct pits behind the ears. and had a clear account, from the father, nurses, &c., of the three days' previous fever (in the former attack) and illness, of the appearance and progress of the pustular eruption; the pustules about 130 in number. The child they alleged to have then caught the disease from a person in the house very ill of variola.

Patients having the varioloid disease, and communicating unmodified variola to the unprotected, and the subjects of primary variola, whether the disease were mild or severe, producing in the inoculated or vaccinated the mild varioloid disease, were circumstances so common, that it is hardly necessary to mention individual instances.

In three apartments of a house with a common stair, I saw, belonging to three families, ten children in different stages of variolous or varioloid diseases. Here an unprotected child had accidentally contracted variola, and died. From this child the mother inoculated three others, two of whom had the disease mildly, but with a numerous eruption; and the third so severely as to cause the child's life for a time to be despaired of. The vaccinated children of the other two families were running about in the desiccating stage of a very mild varioloid. In this house only one vaccinated child resisted altogether the contagion.

The son and daughter, aged respectively sixteen and seventeen, of a tradesman, being exposed to variolous contagion, contracted the varioloid disease, and went through it favorably, suffering only from the invasive fever; but both had a copious eruption, which left elevations and copper-coloured marks, that remained for months. Whilst under the eruption, they were visited by a druggist's apprentice, who, at the same time with them, had been vaccinated by his father, a surgeon. About ten days after these visits, the apprentice was seized with unmodified variola, of which he died.

A woman, aged twenty-eight, the wife of a tailor, contracted variola, and unexpectedly recovered. During her illness, the husband, aged thirty, who had had variola when two years of age, and who remained pitted in the face, attended upon her, and slept in a bed in the same small room. On the tenth day of his wife's disease, he became feverish, and continued so for two days, when a pretty numerous eruption appeared. This went through the varioloid course, and after the third day the patient walked about. In this family there was only one child, four years of age: he had been vaccinated, and he resisted the contagion, though constantly with his father and mother.

The men belonging to the ships-of-war that were at Halifax during the summer and winter, did not entirely escape. In the early part of October, a man, twenty-seven years of age, lately arrived from New Brunswick, and only a week before entered as a volunteer on board the Hussar, (after residing in Halifax a fortnight,) was seized with variola. On the first appearance of eruption, he was sent to the hospital. The disease proved confluent, and he died on the thirteenth day. On examination at entering, he positively stated that he had had the smallpox, as he had been assured by his mother; yet, on becoming ill, his confidence in this assertion seemed diminished.

The occurrence of this case rendered a particular examination of every person belonging to the ship necessary. From this inquiry it was found that, of 301 persons in or attached to the ship, 163 had had natural variola, 112 remaining pitted, and 51 not pitted; 88 had been inoculated, 7 remaining pitted, and 81 not pitted; 32 had been vaccinated, chiefly young officers and boys; 6 vaccinated or inoculated; 2 vaccinated or inoculated; and 10 remained who had no knowledge of having been inoculated or vaccinated, or of having had variola. These ten were immediately vaccinated, but only in four was the disease produced, the others resisting the virus, though the operation was in each six times repeated.

The account given during this examination by two marines satisfied me that at a former period they had had mild variola, notwithstanding preceding good vaccinia; and there could be no doubt that one of the clerks, subsequent to inoculation so effectual that several pits remained, had had variola very severely, remaining greatly pitted.

On board the Alligator two men caught the disease: the first, aged twenty-one, was sent to the hospital, on the 27th November, with a mild varioloid disease, though the pustules were numerous. On his left arm there were two large good foreolous cicatrices, more resembling the scars after inoculation than vaccination, but he did not know whether he had been inoculated or vaccinated: that one or the other had been done, he was certain.

Six days after this man, the second, aged twenty-four, was sent to the hospital. This case proved confluent, was attended by delirium, the pustules never matured, and he died on the seventh day of the eruption. This patient stated, distinctly and positively, that, when fourteen years of age, he had had smallpox, which confined him to bed for three weeks.

The Ringdove being appointed to winter at Halifax, before she sailed from Bermuda, a careful investigation was instituted regarding smallpox, and the unprotected were vaccinated as soon as lymph could be obtained. Notwithstanding this, not long after her arrival, smallpox appeared on board: three were injected, and of these one died. The subject of this fatal case had been inoculated; he had a good scar on the arm, and some variolous pits.

Notwithstanding the occurrences narrated, vaccination was still anxiously sought for where it had not been previously had, and renewed, or at least reattempted, in those who had been previously vaccinated; as much (indeed, I believe more,) from the almost certainty of its procuring a mild varioloid disease, should the person contract the smallpox, as to protect from the wide spread of variolous contagion.

---

